# Enhance the performance of photovoltaic solar panels by a self-cleaning and hydrophobic nanocoating

**DOI:** 10.1038/s41598-022-25667-4

**Published:** 2022-12-08

**Authors:** Samir Ahmed Tayel, Ashour Eid Abu El-Maaty, Eman Mohamed Mostafa, Youssef Fayez Elsaadawi

**Affiliations:** 1 Faculty of Agricultural Engineering, Al-Azhar University, Cairo, Egypt; 2 Faculty of Agricultural Engineering, Al-Azhar University, Assiut, Egypt; 3 Egyptian Petroleum Research Institute, Cairo, Egypt

**Keywords:** Renewable energy, Engineering, Nanoscience and technology

## Abstract

The photovoltaic (PV) solar panels are negatively impacted by dust accumulation. The variance in dust density from point to point raises the risk of forming hot spots. Therefore, a prepared PDMS/SiO2 nanocoating was used to reduce the accumulated dust on the PV panels' surface. However, the effectiveness of these coatings is greatly influenced by geographical and climatic factors. Three identical PV modules were installed to run comparable experimental tests simultaneously. The first module is coated with the prepared PDMS/SiO_2_ nanocoating, the second is coated with commercial nanocoating, and the third module is uncoated and serves as a reference. The prepared nanocoating was hydrophobic and had a self-cleaning effect. The fill factors for the reference panel (RP), commercial-nanocoated panel (CNP), and prepared-nanocoated panel (PNP), were 0.68, 0.69, and 0.7, respectively. After 40 days of exposure to outdoor conditions, the dust densities on the RP and PNP panels' surfaces were 10 and 4.39 g/m^2^, respectively. Thus, the nanocoated panel's efficiency was found to be higher than that of the reference panel by 30.7%.

## Introduction

Solar radiation can be divided into three main wavebands: Ultraviolet (UV) radiation for wavelengths below 400 nm (photons with energy greater than 3.1 eV). Visible (VIS) radiation for wavelengths between 400 and 760 nm (photon energy between 1.6 and 3.1 eV). Infrared (IR) radiation for wavelengths greater than 760 nm (photon energy below 1.6 eV). The near infrared (NIR) ranges up to 4 m^1^. Egypt has high solar irradiance with annual global irradiance exceeding 2000 kWh/m^2^^2^. The optimal orientation of a solar conversion system is towards the equator, yielding an orientation to the south in the northern hemisphere (azimuth angle = 0); and an orientation to the north in the southern hemisphere (azimuth angle = 180). The optimal tilt angle is affected by the latitude of the location and the day of the year^3^. In Egypt, the optimal tilt angle of PV modules and collectors to maximize captured solar energy is β_opt_ = φ ± 15°^4^. Solar technology is currently the third most widely used renewable energy source in the world after hydropower and wind power. Furthermore, electricity from fossil fuels causes CO_2_ emissions of between 400 g and 1000 g CO_2_/kWh, while the CO_2_ emissions from silicon-based solar panels are negligible^5^. The parameters provided by PV module manufacturers are measured in standard test conditions (STC). Such circumstances, though, are uncommon in the field. The experimental measurement of the I–V characteristics is of great importance since it can serve as proof of the quality and performance of every PV system. The short circuit current (Isc) and the open circuit voltage (Voc) are the key properties of the I-V and P-V curves. For each point on the IV curve, the product of current and voltage represents the power output at that operating condition. The fill factor (FF) is defined as the ratio of the product of Pm and Isc Voc, which defines the squareness of the curve^6^. The easiest way to measure and plot an I–V curve is by using a resistive load. It consists of a combination of power resistors with multiple resistance values, switched gradually from a small to a high resistance value for short periods of time. Each resistor value is considered an operating point on the I–V curve^7^. Radiation loss due to dust accumulation reduces PV output power. The variable dust accumulation at any point on the PV surface results in a different distribution of sunlight entering the PV array, increasing the possibility of a hot spot that damages the PV panels^8^. Higher dust density reduces PV short-circuit current, open-circuit voltage, and output power. Dust with a density of 10g/m^2^ can reduce the maximum PV output by about 34%^9^. Regular cleaning of PV modules is essential to maintain their performance. Several PV module cleaning techniques are available and can be classified as manual, automatic, or self-cleaning. The main problem with manual cleaning is the high consumption of water and electricity. The automated process also requires power, and the initial cost is very high. Therefore, self-cleaning methods such as hydrophobic coatings are good options for maintaining PV modules. The coating process does not require electricity to operate and does not damage panels while cleaning. This process is more reliable and cheaper^10^. It has been well established that the use of nano-fillers such as nano-silica, titanium dioxide, zinc oxide, etc. can create hydrophobic coatings for large-scale industrial applications. By definition, hydrophobic nanocoating’s contain at least one nano-sized component that plays a central role in the coating properties, or nano-scale hydrophobic coating's morphology^11^. The use of a commercial hydrophobic SiO_2_ coating nanomaterial improved the overall performance of the solar PV modules. The output power, which indicates the overall efficiency of the PV system, was increased by 15% compared to the dusty modules and by 5% compared to the uncoated modules that were cleaned manually every day. The overall efficiency of the solar PV modules was increased due to their ability to remove dust without using any energy source^12^. Two PV modules were installed in order to run comparable experimental tests at the same time. The first module is coated with SiO_2_ nanoparticles, while the second is uncoated and serves as a control. A micro-cloth was used to coat the cleaned glass with the prepared nanoparticle solution. The contact angle is approximately 106.02°. According to the Wenzel-Baxter definition, this angle is considered hydrophobic. The average electrical efficiency of the coated module is approximately 13.79%, while that of the uncoated module is approximately 13.29%. It was discovered and concluded that coated panels generate 13% more output power even when the surface is not cleaned on a regular basis^13^.

The main contribution of this work is to enhance the performance of PV solar panels by reducing the dust accumulation on the panels' surfaces over time, thereby reducing cost, effort, and water consumption while cleaning, using PDMS/SiO_2_ hydrophobic nanocoating. monitor the performance after nanocoating in harsh outdoor conditions, represented by the high temperature and dust content during the harvest and summer season in an agricultural environment. Applying nanocoating to the solar panel by spraying with a compressor, which is the method that can be used commercially on a large area of the panels, unlike previous studies that applied nanocoating using a piece of cloth, or by dip coating^13^. Making a comparison between the prepared nanocoating, the commercial nanocoating, and the reference uncoated panel, which showed the good properties and high efficiency of the prepared nanocoating.

## Materials and methods

The metal oxide nano-coating was prepared at the Egyptian Petroleum Research Institute, Nasr City, Cairo, Egypt. The outdoor experiments were carried out in Itay al Barud, Beheira Governorate, Egypt, located at latitude 30.529264° N, longitude 30.4213071° E, and 6 m above sea level. The nanocoating characteristics (chemical and physical) analyses were carried out in the National Center for Research, the Egyptian Petroleum Research Institute, and the Faculty of Science, Alexandria University's electronic microscope unit.

### The photovoltaic system

The photovoltaic system consists of three main components; PV panels, charging controller, 12v 9A.h. battery, DC pump, and other electrical components (such as wires and MC4).

### Photovoltaic panels

**Figure 1:**
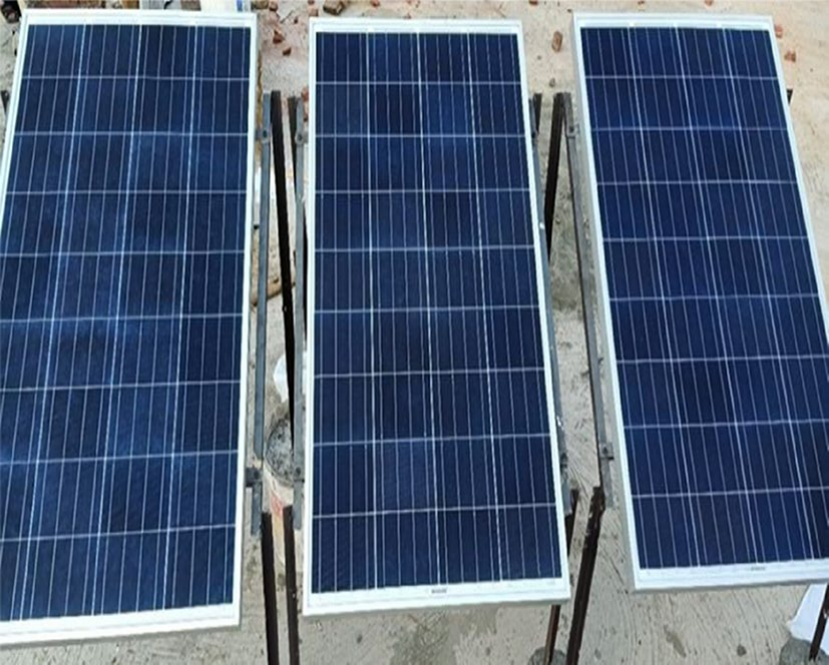
The Pv panels.

Three panels were used to generate power to operate the pumping system. Each panel has a rated power of 100 W as shown in Fig. 1 and datasheet in Table 1.

**Table 1:** PV panel data sheet.

Model type	ESP-100 PPW
Max power (P Max)	100 W
Voltage at max power point (Vmp)	180 V
Current at max. power point (imp)	5.56 A
Open circa voltage (Voc)	21.6 V
Short circuit current (Isc)	6.11 A
Normal operating temp. (NOCT)	45 °C ± 2 °C
Panel dimension (mm)	1009 × 670 × 40
Panel weight (kg)	8 (approx.)
Max. series fuse	15 A
Max. system voltage	1000 VDC (EC)
Module efficiency STC	15.5%

### Solar charging controller

**Figure 2:**
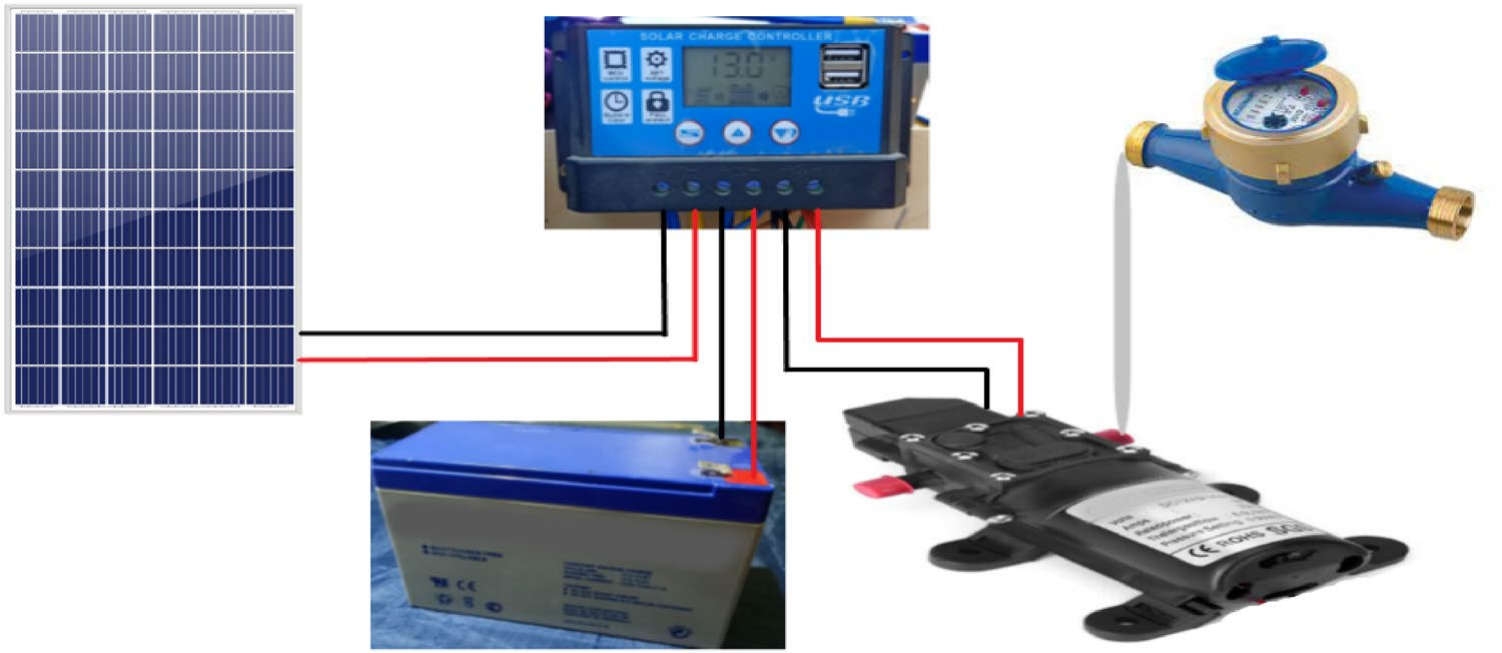
PV system block diagram:

The solar charge controller is used to charge the battery by regulating and controlling the output of the solar panels; It also protects the battery from overcharging or over discharging. A PWM 12v, 10 A solar charging controller is shown in Fig. 2 and its datasheet in Table 2.

**Table 2:** Solar charging controller data sheet.

Model	YJSS10
Rated voltage	12 V/24 V auto
Charge current	10A
Discharge current	10A
Max solar voltage	< 50 V
Low voltage disconnect	10.7/21.4 V
Low voltage reconnect	13.7/27.4 V
Standby current	< 15 Ma
Charging mode	PWM
Working temperature	−20 ℃ to + 60 ℃
Size(L × W × H)	133 × 70 × 30 mm
Weight	150 g

### Sealed lead acid battery

Batteries are commonly used in photovoltaic systems to store energy generated by photovoltaic panels during the day and supply electrical loads when needed (at night or on cloudy days). In addition, batteries are also needed to operate the solar charge controller and off-grid inverter. The Ultracell UXL9-12 battery is shown in Fig. 2 and its datasheet in Table 3.

**Table 3:** The Ultracell UXL9-12 battery datasheet.

Nominal voltage	12 V
Nominal capacity	9 Ah
Internal resistance	19 mΩ
Operating temperature	−15 to 50 co
Design floating life at 20 co	15 years
Initial charging current	˂ 2.7 A

### The DC water pump

12VDC, 72 W The self- priming high-pressure diaphragm water pump shown in Fig. 2 was used as a DC load for PV panels, and the pump datasheet illustrated in Table 4.

**Table 4:** DC water pump datasheet.

Type	Good pumps
Rated power (W)	72 W
Voltage	12 V
Current	6 A
Pressure	0.90 MPA
Size	16.5 × 10 × 6.2 cm
Flow capacity	6 L/min
Weight	Approx.600 g

### The PV system diagram

The PV system block diagram and the connections of its components are illustrated in Fig. 2.

### Measurement devices

**Figure 3:**
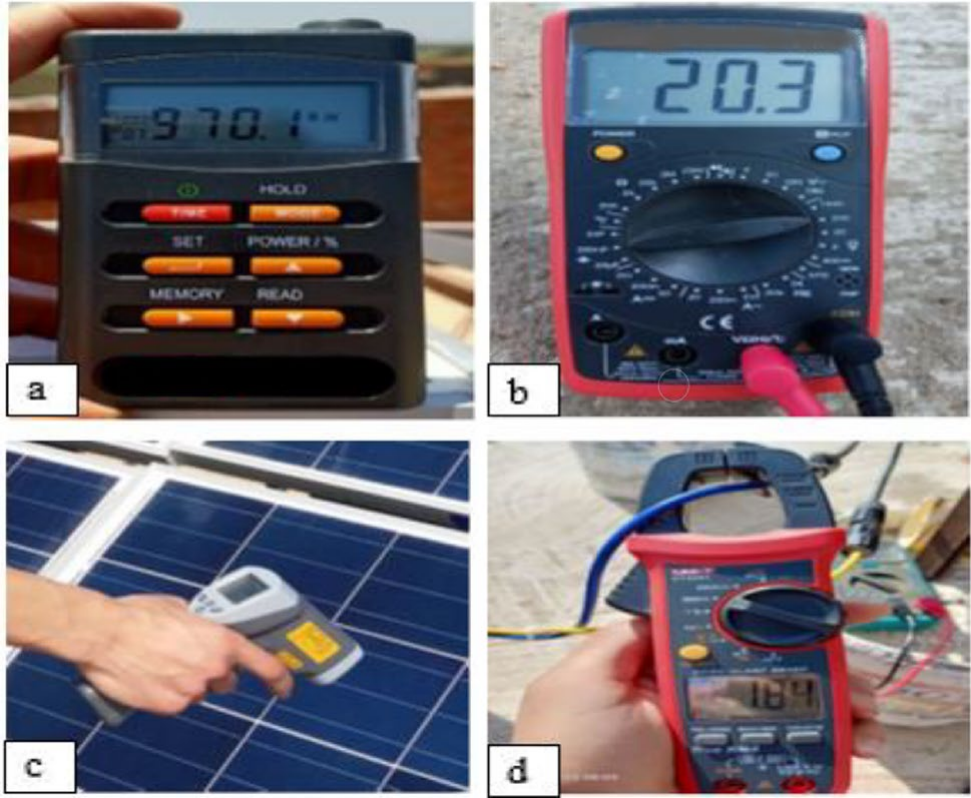
Measurement devices.

The ISM 400 solar radiation meter was used to measure irradiance in W/m^2^ (Fig. 3a). The digital multimeter was used to measure voltage (V), current (A), and resistance (Ω) (Fig. 3b). The digital clamp meter was used to measure current in a conductor without making physical contact with it (Fig. 3c). The digital infrared thermometer (DT8011T) was used to measure the PV panel surface temperature (Fig. 3d).

### Methodology

The nanocoating preparation experiments were conducted during the period from April 2021 to May 2022, and the outdoor experiments during the period from May 2022 to July 2022. The measurements were taken during the day from 8 a.m. to 4 p.m. every hour. The measurements included solar radiation, PV panel's surface temperature, PV panel's output (DC current, DC voltage), pump's discharge, pressure, dust accumulation density g/m^2^, and I-V characteristics. The nanocoating characteristics, chemical and physical analyses (contact angle, light transmittance, measuring molecules' size, components percent of nano-coating, atomic photographing) were conducted in the atomic microscope unit in the National Center for Research, Egyptian Petroleum Research Institute, and Faculty of Science, Alexandria University.

### Preparation of hydrophobic nanocoating

#### Synthesis of PDMS/SiO_2_ nanocomposites

The Polydimethylsiloxane (PDMS) precursor Part-A (Sylgard184 elastomer base, 3 gm). Has been mixed with toluene and anhydrous ethanol. Then, add 20 ml of sodium silicate 5%(w/v) mixed with PDMS, 80 ml of ethanol absolute, and 2 ml of ammonium hydroxide NH_4_OH, then stir with a magnetic stirrer at 30–35 °C for 2 h. After that, leave the sample for 24 h. The pale white solid product obtained was centrifuged at 4000 rpm for 10 min after thorough washing with double distilled water to remove all the ions, and then centrifuged at 4000 rpm for 10 min and dried at 70 °C for 2 h.

### Preparation of transparent hydrophobic nanocoating

Samples were prepared via the following procedures: the PDMS/SiO_2_ nanocomposites were mixed with ethanol, isopropanol, and its curing agent (the weight ratio of PDMS/SiO_2_ nanocomposites to curing agent was 10:1 for samples). Then, the mixture was dissolved homogeneously with the help of an ultrasonic washer (29 kHz, 150 W) for about 30 min. Then, the prepared nanocoating was applied on the PV panel by spray coating.

### Solar irradiance measurement (W/m^2^)

The intensity of solar radiation (irradiance W/m^2^) was measured every hour using a digital solar radiation meter. The irradiance meter was tilted at an angle equal to that of the solar panels (15°) as shown in Fig. 3a.

### Solar panels output (P_PV_, η_PV_, FF)

The electrical output power was calculated from watts' law according to Eq. (1)^14^.

1 $${\mathrm{P}}_{PV}=\mathrm{V}*\mathrm{I}$$

where, V is the PV panel voltage (V), and I is the PV panel current (amp.).

The efficiency of the PV panels (η_pv_) was calculated as a ratio of the PV panels' output power and the input solar power (Eq. 2).

2 $${\eta }_{PV}=\frac{V * I}{A * G}$$

where, A is the PV panel surface area (m^2^), and G is the intensity of solar radiation (W/m^2^).

FF is determined by the quotient of the peak power (Pmp), and the theoretical maximum power obtained from the product of the open-circuit voltage Voc and the short-circuit current Isc, It is calculated from Eq. (3)^15^.

3 $$FF=\frac{{V}_{mp}*{I}_{mp}}{{V}_{oc}*{I}_{sc}}= \frac{{P}_{mp}}{{V}_{oc}*{I}_{sc}}$$

where, FF is the fill factor.

### I-V and P–V curves plotting method

**Figure 4:**
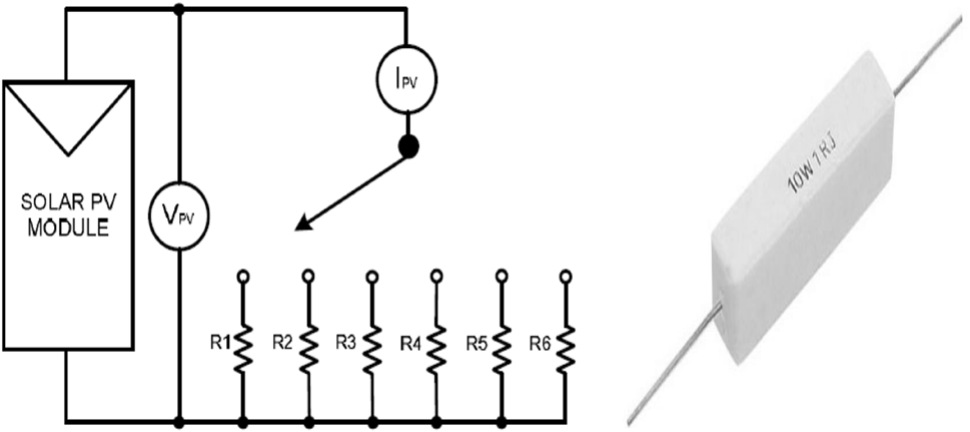
The varying resistance load and the 10 W power resistor.

Many parameters and values can be obtained from these curves as Voc, Isc, Vmp, Imp, Pmp, and FF. The PV panel's characteristics can be changed by varying the load resistance (Fig. 4) that is connected to the PV panel^16^. By increasing the load resistance, the values of the output voltage and current from the module were changed from 0 V to Voc, and from Isc to 0, respectively^7^. The I–V curve can be obtained by plotting voltage and current, and the P–V curve can be obtained by plotting voltage and calculated power. A combination of power resistors with different resistance values from 0.10 to 24 Ω with a 0.5 Ω increase in every measurement was used as a variable resistance (Fig. 4).

## Results and discussion

### Nanocoating characterization

#### Scanning electron microscopy (SEM)

**Figure 5:**
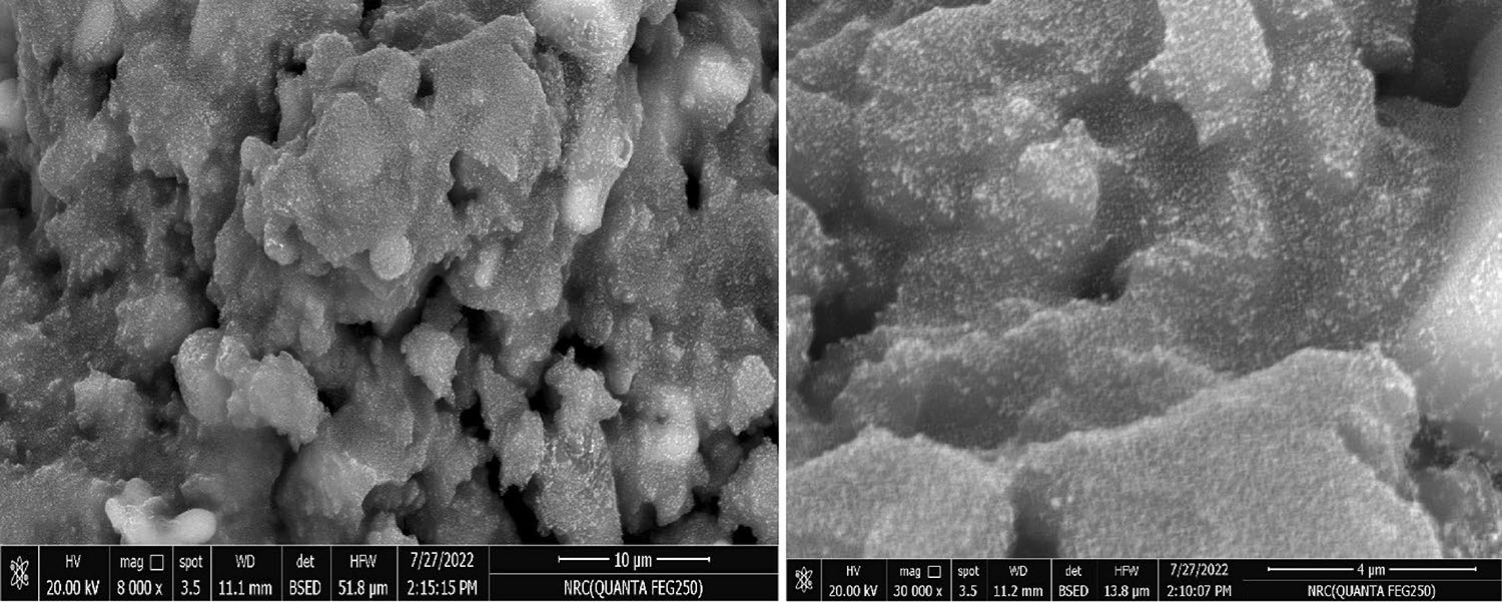
SEM images of PDMS/SiO2 nanocoating.

The surface morphology of the nanocoating was observed with a Quanta FEG250 scanning electron microscope. Representative images of the sample with low (8000×) and high (30,000×) magnification are Fig. 5. The SEM images show the surface roughness of the nano-coating, which is an important factor for the hydrophobicity and thus the contact angle. The nanocoating has increased the surface roughness at the nano and micro scale, and this increases the hydrophobicity and contact angle according to the Wenzel and Cassie models^17^.

### Energy dispersive X-ray (EDX)

**Figure 6:**
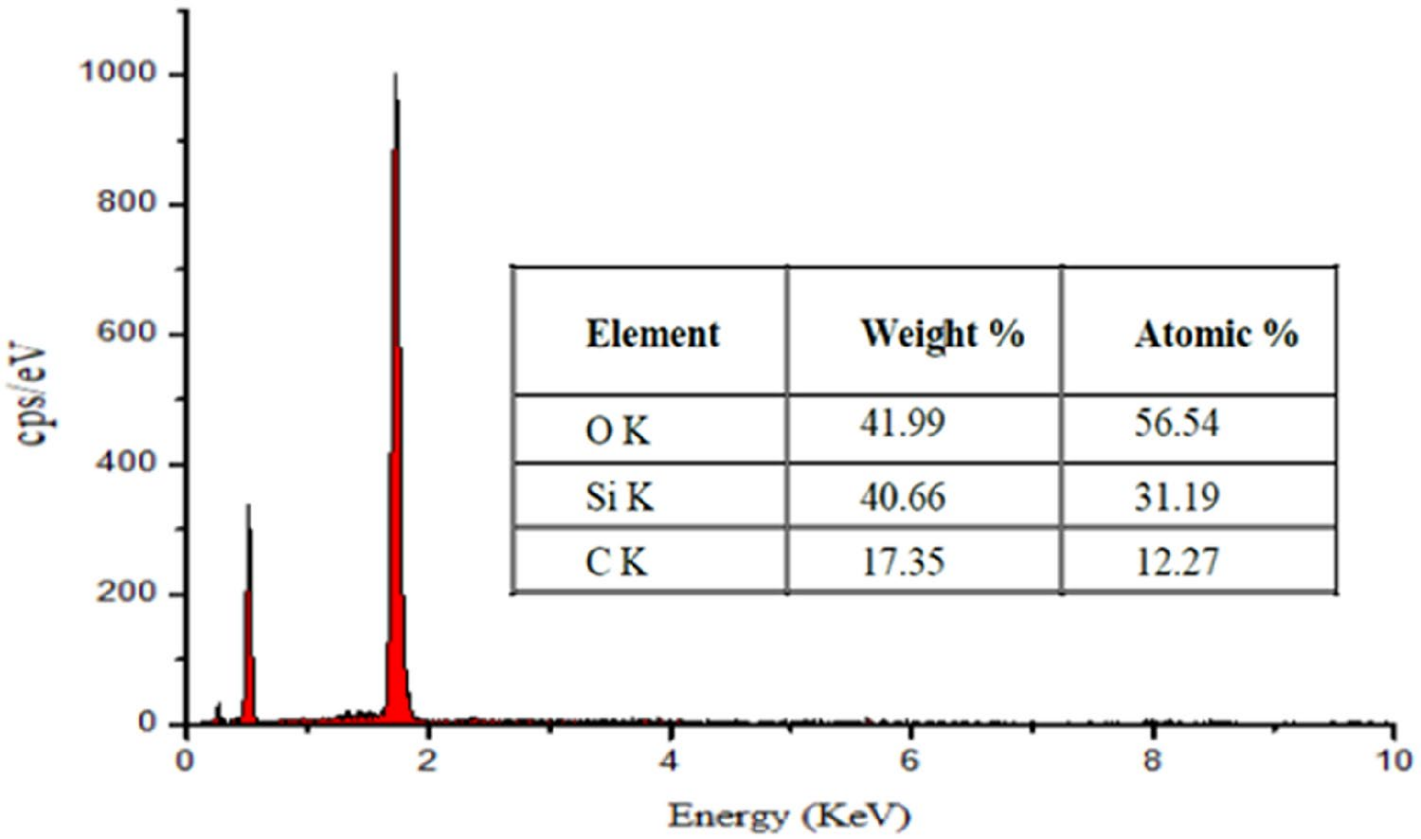
Energy dispersive spectroscopy (EDS) for nanocoating.

Figure 6 shows the EDX spectrum for nanocoating. The presence of Si and O, which suggests the proper dispersion of silica nanoparticles throughout the coating^18^, is undeniably supported by EDX data, and the presence of C, along with O, accounts for the functionalizing chemicals used. The weight percent of O, Si, and C was 41.99, 40.66, and 17.35%, respectively.

### Transmission electron microscopy (TEM)

**Figure 7:**
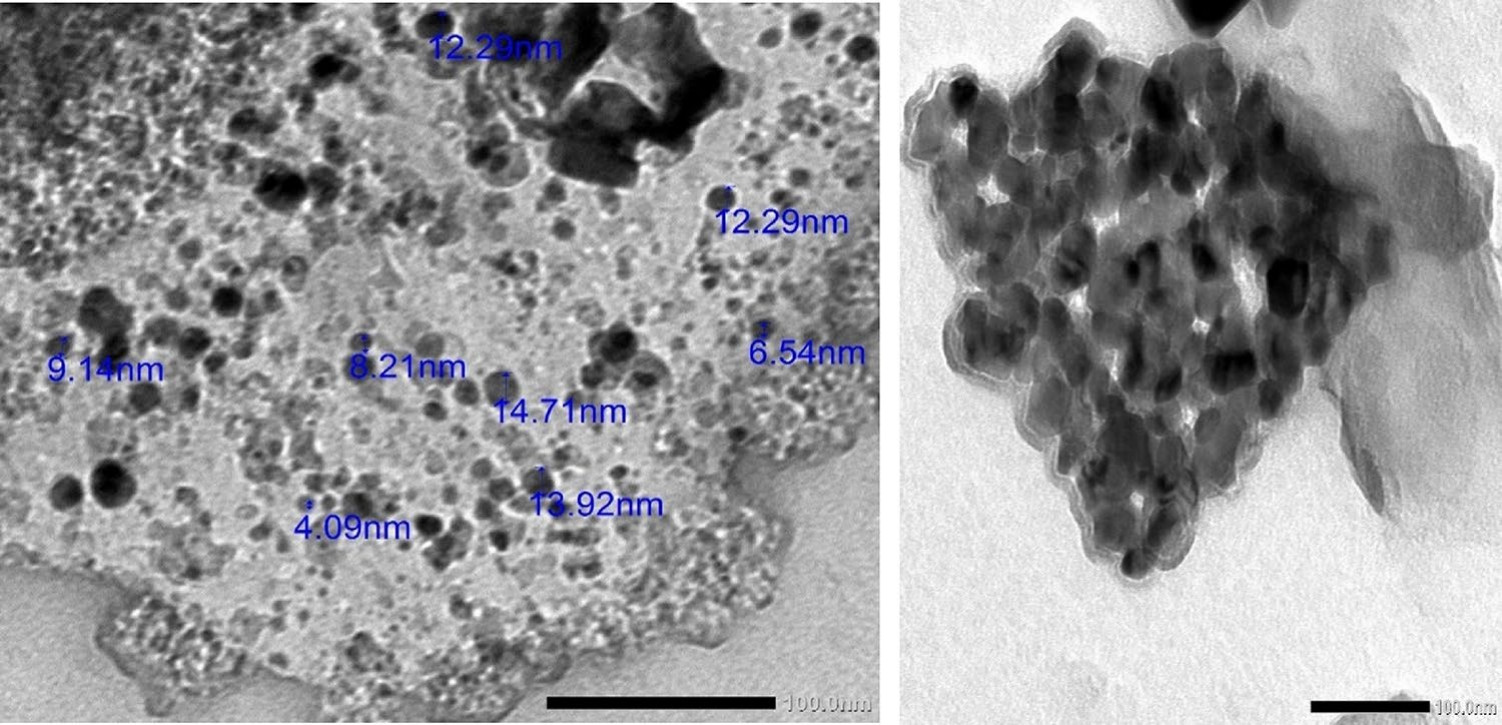
TEM images of the PDMS-SiO_2_ nano coating.

Transmission Electron Microscopy (TEM) is a crucial technique for determining the NP's structure, size, and distribution pattern^19^. The metal oxide nanoparticles are well dispersed in the PDMS polymer. The average nanoparticle size was 11 nm as shown in (Fig. 7). Through the hydrophobic chain of PDMS, PDMS-SiO2 nanoparticles cross-link with one another, which further leads to the formation of clusters of different sizes and then the micro-nanostructure. as shown in Fig. 7. The average cluster size was 80 nm. All TEM images magnifications were 100 nm.

### Ultraviolet–visible spectrophotometer (UV–Vis):

**Figure 8:**
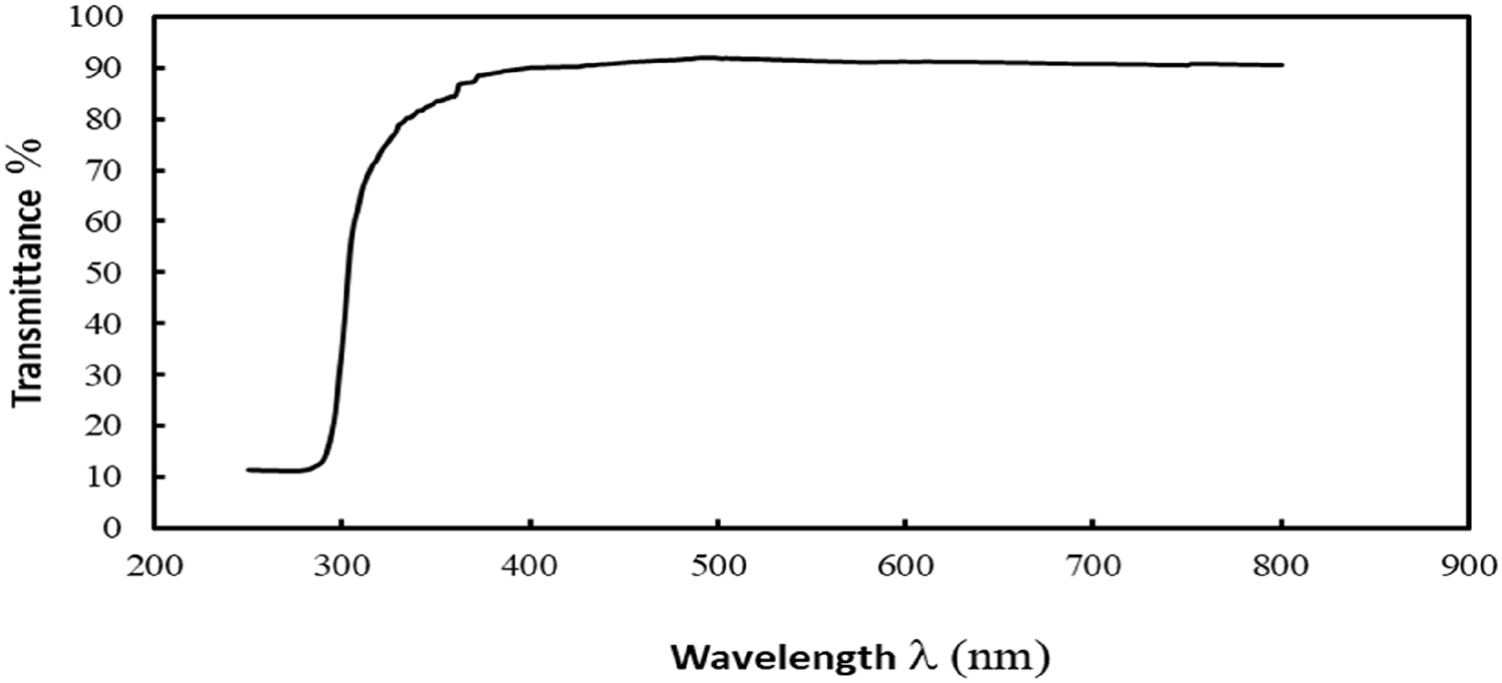
The UV–Vis spectroscopy of the PDMS/SiO_2_ nanocoating.

The Uv–Vis spectroscopy curve in illustrates that the nanocoating had a high transmittance in the visible light range (Fig. 8). The average transmittance for the prepared nano coating was 91% in the visible light range (400–800 nm) and the nanocoating was resistant to UV (200–390 nm) radiation.

### Fourier transform infrared spectroscopy (FT-IR)

**Figure 9:**
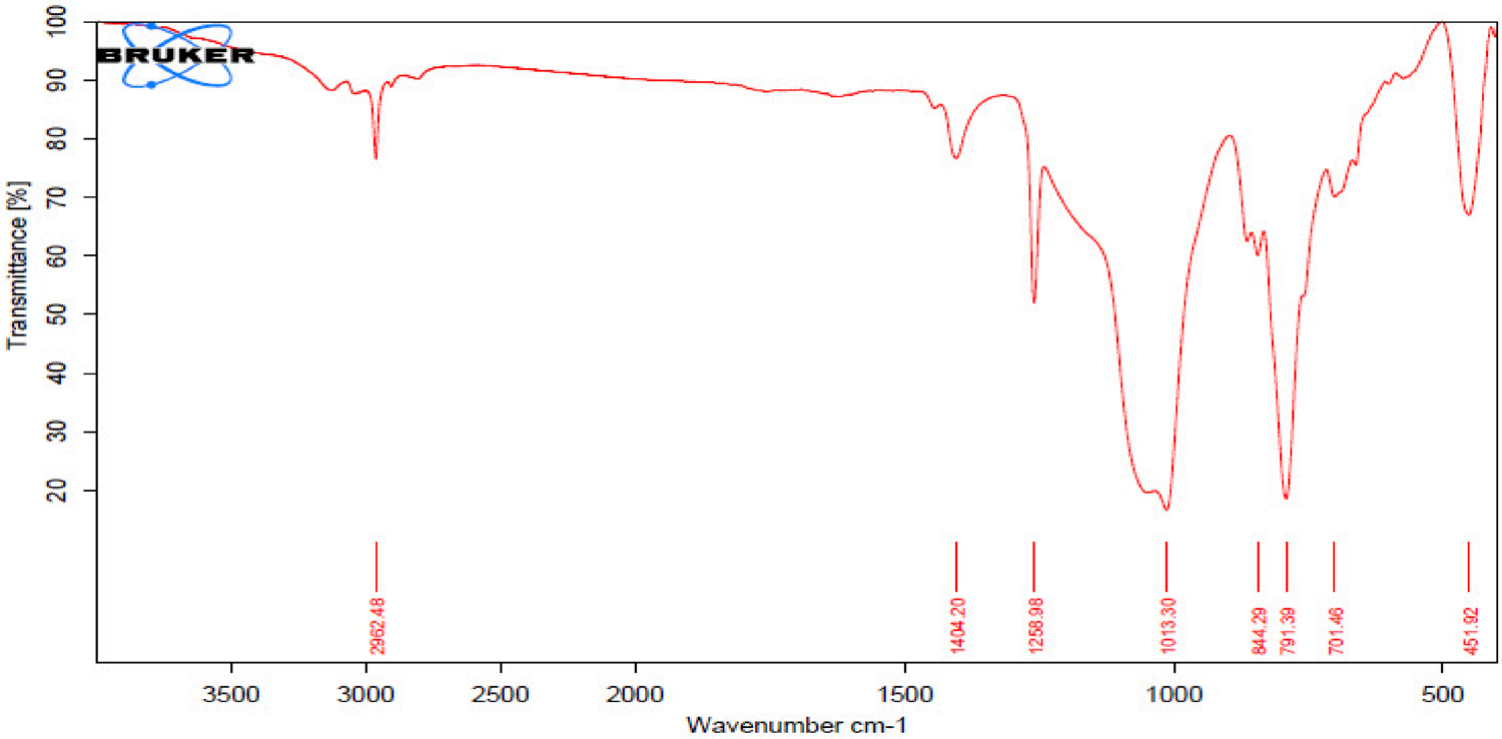
Fourier Transform Infrared Spectroscopy for PDMS/SiO_2_ nanocoating.

Fourier transform infrared spectroscopy (FTIR) is a technique for identifying distinctive functional groups from spectral bands, allowing us to determine the conjugation between the nanomaterial and the adsorbed biomolecule^20^. The analysis is determined by measuring a sample's absorbance to an incident infrared spectrum between 400 and 4000 cm^−1^ (Fig. 9). The major spectral bands and the characteristic functional groups of the spectral bands are shown in Table 5.

**Table 5:** The characteristic functional groups of the FTIR spectral bands.

Frequency	Appearance	Group	Frequency	Appearance	Group
2962.48	Medium	Si–CH_3_	844.25	Strong	C–H
14,042	Medium	O–H	791.39	Strong	Si–C
1258.98	Strong	Si–C	701.46	Strong	C=C
1013.3	Strong	Si–O–Si	451.92	Strong	Si–O–Si

### Surface wettability of nanocoating (WCA)

**Figure 10:**
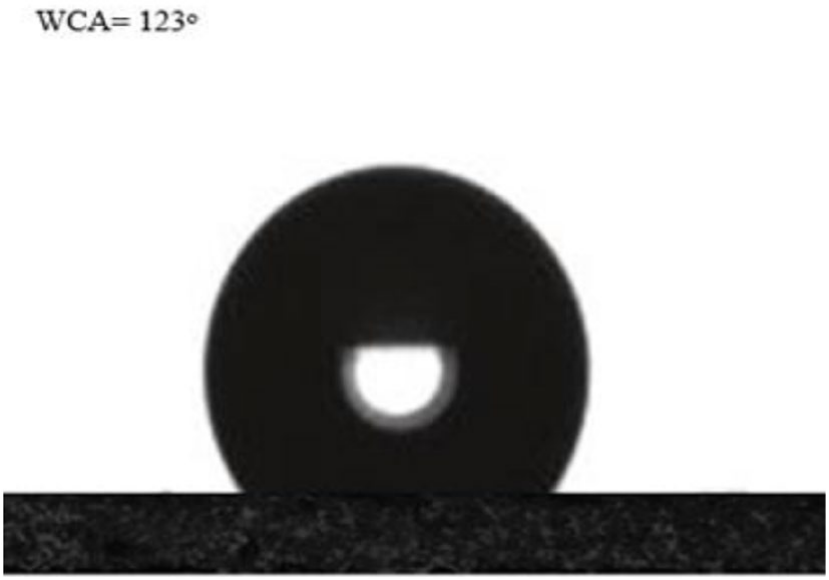
Water contact angle of PDMS/SiO_2_ nanocoating on glass substrate.

The contact angle, which varies from 0° to 180°, can be used to qualitatively identify whether a surface is hydrophilic or hydrophobic. The contact angle is a measurement of the relative magnitudes of adhesive (liquid to solid) and cohesive (liquid to liquid) forces acting on a liquid. Contact angle measurement is probably the method used the most frequently to determine solid surface tension. The three most widely used methods for measuring contact angles are the sessile drop, captive bubble, and Wilhelm plate methods. In the used sessile drop experiment, a droplet of a completely purified liquid is administered to a solid surface using a syringe or a micropipette. A goniometer placed in the eyepiece of a low-magnification microscope is typically used to view the droplet and calculate the resulting contact angle^21^. The contact angle was measured through the manipulation of water drop shapes on the samples using the OCA 15EC Contact angle model produced by the company of Data Physics Instrument Gmbh. The water contact angle for the prepared nano coating was 123 degrees, which means that the PDMS/SiO2 nanocoating is hydrophobic (Fig. 10).

### Current–voltage curves for clean panels (I–V curves)

**Figure 11:**
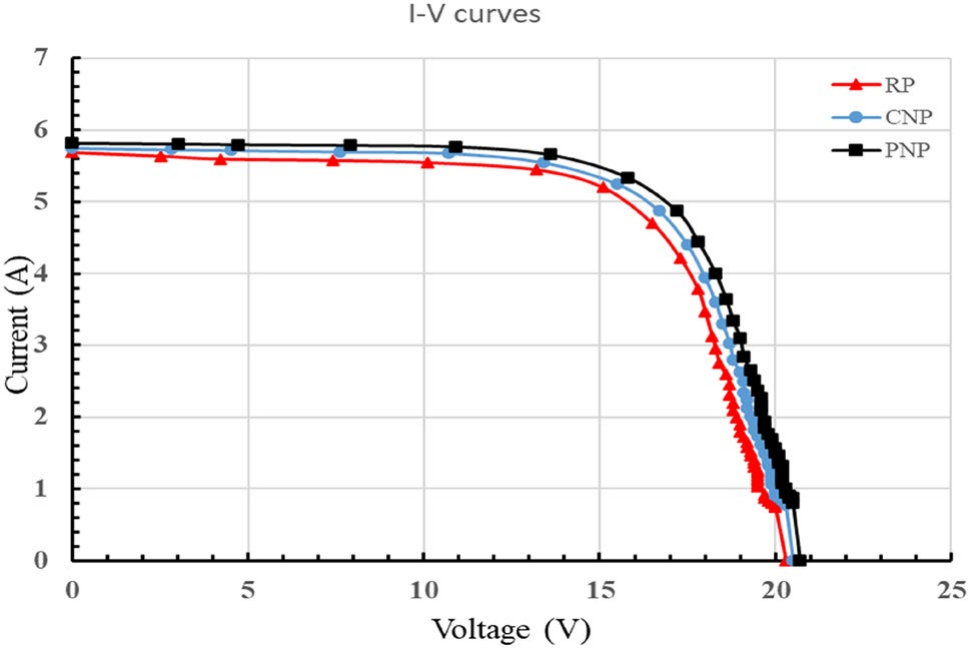
The I–V curve for RP, CNP, and PNP.

The I-V curves for a clean reference panel (RP), a commercial-nanocoated panel (CNP), and a prepared-nanocoated panel (PNP) are shown in Fig. 11 and the important points in Table 6. The short circuit current Isc was 5.69, 5.7, and 5.82 A, respectively, and the open circuit voltage Voc was 20.3, 20.5, and 20.7 V, respectively, at solar radiation of 960 ± 7 W/m^2^ and a PV panel surface area of 0.6 m^2^. The characteristics and efficiency of the prepared nano-coated panel were higher than those of the reference and commercial nano-coated panels. This is due to the roughness and nano-micro scale pyramidal shapes that are widely spread on the surface of the nano-coating, which reduces the reflectivity of light on the surface of the panels^22^.

**Table 6:** Important characteristics for I–V and P–V curves.

Resistance	RP			CNP			PNP		
Ω	V	I	Power	V	I	Power	V	I	Power
Short circuit	0.0	5.69	0.0	0.0	5.75	0.0	0.0	5.82	0.0
2.50	15.1	5.20	78.5	15.5	5.25	81.4	15.8	5.34	84.4
Open circuit	20.3	0.00	0.0	20.5	0.00	0.0	20.7	0.00	0.0

### Power-voltage curves for clean panels (P–V curves)

**Figure 12:**
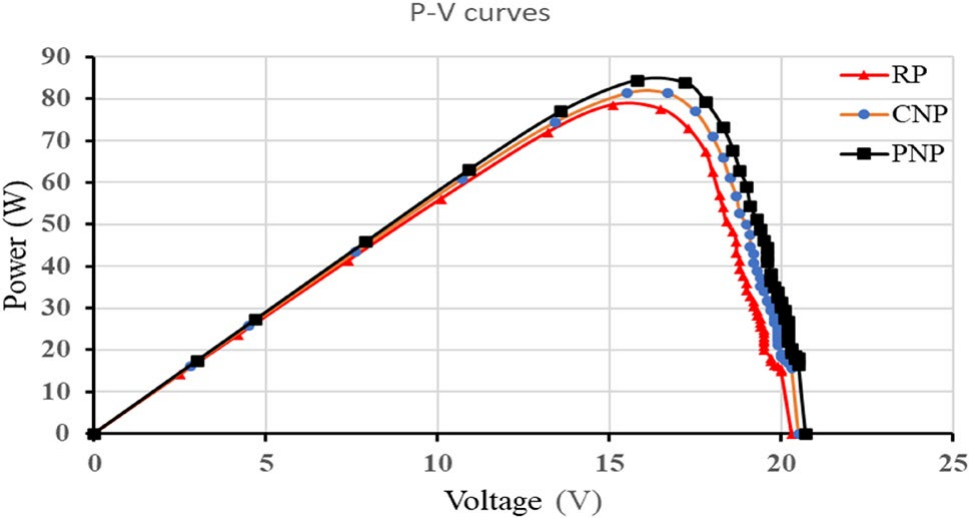
The P–V curves for RP, CNP, and PNP.

The maximum power Pmax for clean reference panel (RP), commercial-nanocoated panel (CNP), and prepared-nanocoated panel (PNP), was 78.5, 81.4, and 84.4 W, respectively, as shown in Fig. 12.

### Fill factor for clean panels (FF)

The fill factors for clean reference panel (RP), commercial-nanocoated panel (CNP), and prepared-nanocoated panel (PNP), were 0.68, 0.69, and 0.7, respectively. The main parameters are illustrated in Table 7. Because the nanocoated panel produces the highest Imp and Vmp, it is the panel with the highest fill factor. This indicates the high efficiency compared to other panels^23^.

**Table 7:** The main parameters for the clean panel's FF.

RP				CNP				PNP			
V_oc_	I_sc_	Isc*Voc	FF	V_oc_	I_sc_	Isc*Voc	FF	V_oc_	I_sc_	Isc*Voc	FF
20.3	5.69	115.5		20.5	5.75	117.9		20.7	5.82	120.5	

Vmp	Imp	Pmax		Vmp	Imp	Pmax		Vmp	Imp	Pmax	
15.1	5.2	78.5	0.68	15.5	5.25	81.4	0.69	15.8	5.34	84.4	0.70

### RP and PNP performance with dust accumulation

#### PV panels' power within 40 days of exposure to external conditions

**Figure 13:**
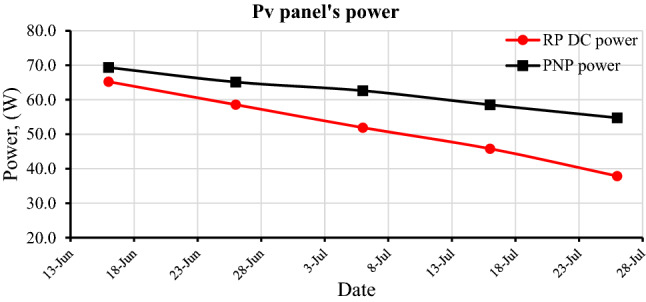
The degradation in the RP and PNP power within 40 days.

The power of the reference panel (RP) and prepared-nanocoated panel (PNP) degrades over time (40 days) due to an increase in dust accumulation density on the panels' surface. The dust acts as a barrier between the sunlight and the photovoltaic cells, trapping a large portion of the sunlight and thus deteriorates the capacity of the solar panels. The power difference between RP and PNP increases with time due to the difference in dust accumulation density on each panel. The RP and PNP average power were 65.2 and 69.4 watts on the first day, 58.6 and 65.1 after 10 days, 51.9 and 62.6 after 20 days, 45.8 and 58.5 after 30 days, and 37.9 and 54.8 after 40 days, respectively, as shown in (Fig. 13). The percentage of power degradation within forty days for RP and PNP reached 42% and 21%, respectively.

### The pumps’ discharge within 40 days of exposure to external conditions

**Figure 14:**
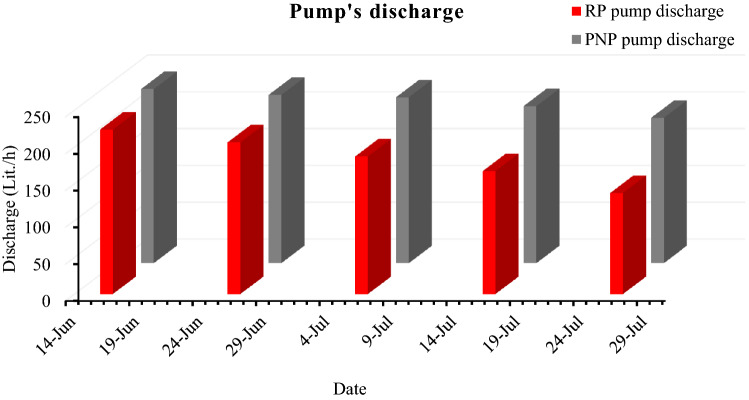
The degradation of RP and PNP pumps' discharge within 40 days.

The degradation in the RP and PNP power leads to the degradation in the discharge of the pumps that are connected to PV panels. The discharge difference between pumps connected to RP and PNP increases due to the increasing difference in output power of each panel with time. The RP and PNP pumps' average discharge were 223.6 and 236.6 L/h on the first day, 206.2 and 228.6 after 10 days, 187.1 and 225.6 after 20 days, 167.2 and 213.5 after 30 days, and 137.4 and 197.7 L/h after 40 days, respectively, as shown in Fig. 14. The percentage of pumps’ discharge degradation within forty days for RP and PNP reached 39% and 16%, respectively.

### Pv panels temperature

**Figure 15:**
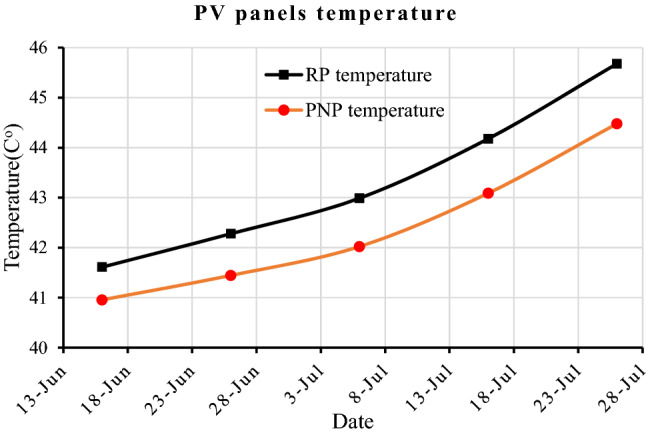
The RP and PNP temperature within 40 days.

The continuous accumulation of dust and dirt on the PV panel surface over time, and the inhomogeneity of the dust density, lead to partial shading on the PV cells, which causes a difference in the solar cells' productivity compared to each other. The low output cells work as a load or resistance to the high output cells. The temperature of the panels increases because of the high internal resistance. The RP and PNP average temperatures were 41.6 and 41.0 c^o^ on the first day, 42.3 and 41.4 after 10 days, 43.0 and 42.0 after 20 days, 44.2 and 43.1 after 30 days, and 45.7 and 44.5 c^o^ after 40 days, respectively, as shown in Fig. 15. The percentage of temperature increasing within forty days for RP and PNP reached 9.85% and 8.5%, respectively. Previous studies found a decrease in efficiency of 0.5%/1 °C^24^. The temperature difference between RP and PNP panels increased with time according to dust density, where it reached 1.2 °C, which degraded the RP panel efficiency by 0.6%.

### Dust density (g/m^2^) and panels' efficiency within 40 days of exposure to external conditions

**Figure 16:**
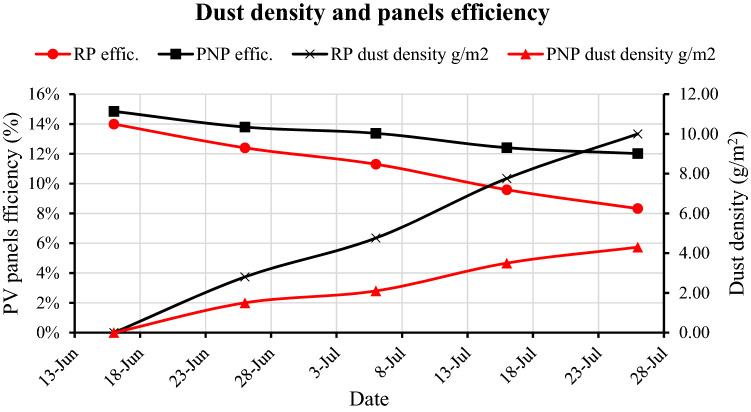
The dust density (g/m^2^) and panels' efficiency (%) within 40 days.

The efficiency of solar panels gradually decreases over time because of the increased density of dust accumulation on the surface of those panels. A large difference occurs between the efficiency of RP and PNP with time due to the difference in the density of dust on each panel. The RP and PNP average dust density were 0.00, 0.00 g/m^2^ on the first day, 2.80, 1.50 after 10 days, 4.76, 2.10 after 20 days, 7.76, 3.50 after 30 days, and 10.00, 4.30 g/m^2^ after 40 days, respectively. This is due to the self-cleaning property of nanocoating, which reduces the amount of dust accumulated on the PNP surface. The RP and PNP average efficiency were 13.99%, 14.85% on the first day, 12.40%, 13.79% after 10 days, 11.30%, 13.38% after 20 days, 9.59%, 12.41% after 30 days, and 8.32%, 12.01% after 40 days, respectively, as shown in Fig. 16.

### RP and PNP performance after self-cleaning:

**Figure 17:**
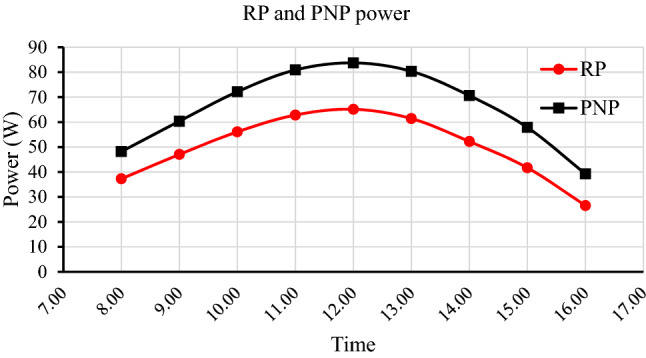
The RP and PNP power after self-cleaning with water.

After 40 days of exposure to weather conditions and dust, a volume of 4 L of water was sprayed onto the surface of each panel in 2 min, through orifices with a 0.5 mm diameter, to test the self-cleaning property. The dust density on RP and PNP before water spray was 10 and 4.30 g/m^2^, while the dust density after water spraying was 4.80 and 1.12 g/m^2^ respectively. As a result of hydrophobicity and consequently the low surface energy for nanocoating^25^, the dust was removed by water droplets by 74% in the nanocoated panel (PNP), compared to 52% for the uncoated reference panel (RP). RP and PNP had average powers of 50.03 and 65.93 W, respectively (Fig. 17).

**Figure 18:**
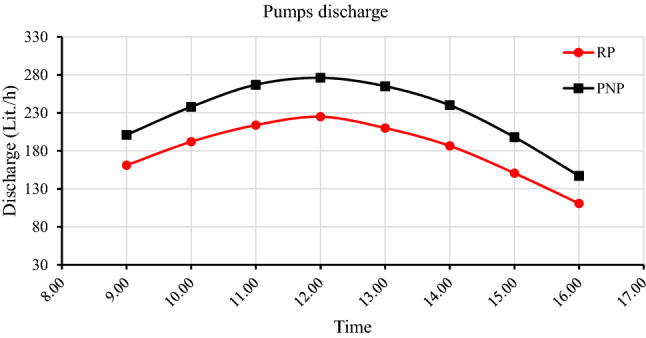
The RP and PNP discharge after self-cleaning with water.

The average efficiency was 11.13% and 14.5%, respectively. The hourly average pump discharge was 181.2 and 229.0 L/h, respectively (Fig. 18). The panel's temperatures were 43.1 and 41.2 °C, respectively. The fill factor value represents curve squareness and gives an idea of the PV panel's quality. Normal values range from 0.7 to 0.8. For RP and PNP, the fill factors were 0.63 and 0.69, respectively (Table 8). The PNP has greater output power, greater efficiency, and the lowest temperature and dust accumulation density. Because of the hydrophobic and self-cleaning properties of the nanocoated panel, the water droplets rolled off and removed a large amount of dust from the panel surface.

**Table 8:** The panels FF and efficiency after self-cleaning.

RP				η	PNP				η
V_oc_	I_sc_	Isc*Voc	FF		V_oc_	I_sc_	Isc*Voc	FF	
20.0	4.80	96.00			20.2	5.65	114.13		

Vmp	Imp	Pmax			Vmp	Imp	Pmax		
15.0	4.04	60.56	0.63	10.5%	15.1	5.22	78.82	0.69	13.7%

## Conclusion

This study was conducted to enhance the performance of PV solar panels by reducing the dust accumulation on panels' surfaces over time, thereby reducing cost, effort, and water consumption while cleaning, using PDMS/SiO_2_ hydrophobic nanocoating. Based on the results of this study, the following conclusions were obtained:

The performance of PV panels was enhanced by the hydrophobic nanocoating. The nanocoating has a good transmittance in the visible light range (400–800 nm). As a result of hydrophobicity and consequently the self-cleaning property of nanocoating, the accumulated dust density on the PNP after 40 days of exposure to outdoor conditions decreased by 57% compared to the uncoated reference panel. Moreover, the dust was removed by water droplets by 74% of the PNP compared to 52% of the RP. The efficiency of the nanocoated panel was higher than the reference panel by 30.7%. It is found and concluded that the nanocoated panel has greater output power and efficiency compared to the reference panel and the previous studies^13^ due to the PDMS/SiO_2_ hydrophobic nanocoating.

## Data Availability

The datasets used and/or analyzed during the current study are available from the corresponding author on reasonable request.
